# Systematic evaluation of irinotecan-induced intestinal mucositis based on metabolomics analysis

**DOI:** 10.3389/fphar.2022.958882

**Published:** 2022-09-15

**Authors:** Qing-Qing Yu, Heng Zhang, Shiyuan Zhao, Dadi Xie, Haibo Zhao, Weidong Chen, Min Pang, Baoqin Han, Pei Jiang

**Affiliations:** ^1^ Laboratory of Biochemistry and Biomedical Materials, College of Marine Life Sciences, Ocean University of China, Qingdao, China; ^2^ Jining First People’s Hospital, Jining Medical College, Jining, China; ^3^ Department of Laboratory, Shandong Daizhuang Hospital, Jining, China; ^4^ Department of Endocrine, Tengzhou Central People’s Hospital, Tengzhou, China; ^5^ MNR Key Laboratory of Marine Eco-Environmental Science and Technology, First Institute of Oceanography, Ministry of Natural Resources, Qingdao, China; ^6^ Laboratory for Marine Drugs and Bioproducts of Qingdao National Laboratory for Marine Science and Technology, Qingdao, China

**Keywords:** cancer, irinotecan, metabolomics, gas chromatography-mass spectrometry, toxicity

## Abstract

Chemotherapy-induced intestinal mucositis (CIM) is a major dose-limiting side effect of chemotherapy, especially in regimens containing irinotecan (CPT-11). Several studies on the pathologic mechanisms of CIM focused on both the genomics and molecular pathways triggered by chemotherapy. However, systematic evaluation of metabolomic analysis in irinotecan-induced intestinal mucositis (IIM) has not been investigated. This study aimed to comprehensively analyze metabolite changes in main tissues of IIM mouse models. Male ICR mice were assigned to two groups: the model group (*n* = 11) treated with CPT-11 (20 mg/kg daily; i.p.) and the control group (*n*= 11) with solvent for 9 days. Gas chromatography-mass spectrometry (GC-MS) was used to investigate the metabolic alterations in the serum, intestinal, colonic, hepatic, and splenic samples of mice between two groups by multivariate statistical analyses, including GC–MS data processing, pattern recognition analysis, and pathway analysis. Forty-six metabolites, including hydrocarbons, amino acids, lipids, benzenoids, hydroxy acids, and amines, had significant changes in levels in tissues and sera of IIM mouse models. The most important pathways related to the identified metabolites were the glycerolipid metabolism in the colon and aminoacyl-tRNA biosynthesis; glycine, serine, and threonine metabolism; and glyoxylate and dicarboxylate metabolism in the liver. Our study firstly provided a comprehensive and systematic view of metabolic alterations of IIM using GC-MS analysis. The characterizations of metabolic changes could offer profound and theoretical insight into exploring new biomarkers for diagnosis and treatment of IIM.

## Introduction

Chemotherapy-induced intestinal mucositis (CIM) is a major dose-limiting adverse reaction of chemotherapy, especially in regimens containing irinotecan (CPT-11) (Lalla et al., 2014; Peterson et al., 2015). With prevalence of about 80% (Jones et al., 2006; Maroun et al., 2007), CIM hinders the effective use of chemotherapy and reduces the quality of life of patients. Studies on the pathologic mechanisms of CIM have focused on genomics and molecular pathways, including inflammatory reaction (Davis 2000; Angel et al., 2001; Bamba et al., 2003), gut-flora imbalance (Alimonti et al., 2004; Alexander et al., 2017), and ischemia (Yu et al., 2020). These alterations can induce metabolites in target organs in CIM. Therefore, metabolomic analysis plays a major role in the investigation of the pathologic mechanisms of CIM.

Metabolomic analysis may reveal the metabolite perturbations associated with diseases using high-throughput technology for multiple metabolites in biological samples. This strategy can provide the global parameters of metabolic profiles and elucidate the underlying mechanisms of diseases (Rinschen et al., 2019; Geng et al., 2020; Geng et al., 2020; Zhao et al., 2021). Metabolomics is an effective tool for discovering biomarkers, investigating the pathophysiology of diseases, subtyping diseases, and developing specific treatment strategies (Bracewell-Milnes et al., 2017; Rinschen et al., 2019). Mass spectrometry-based metabolomic techniques, including gas chromatography-mass spectrometry (GC-MS), liquid chromatography-mass spectrometry (LC-MS), surface-enhanced laser desorption ionization time-of-flight mass spectrometry (SELDI-TOFMS), and matrix-assisted laser desorption/ionization time-of-flight mass spectrometry (MALDI-TOF-MS), are sensitive approaches for simultaneous analysis of several compounds. In previous studies, we evaluated the metabolic alterations of major tissues in the animal model of several drugs using GC-MS (Geng et al., 2021; Cui et al., 2022).

**FIGURE 1:**
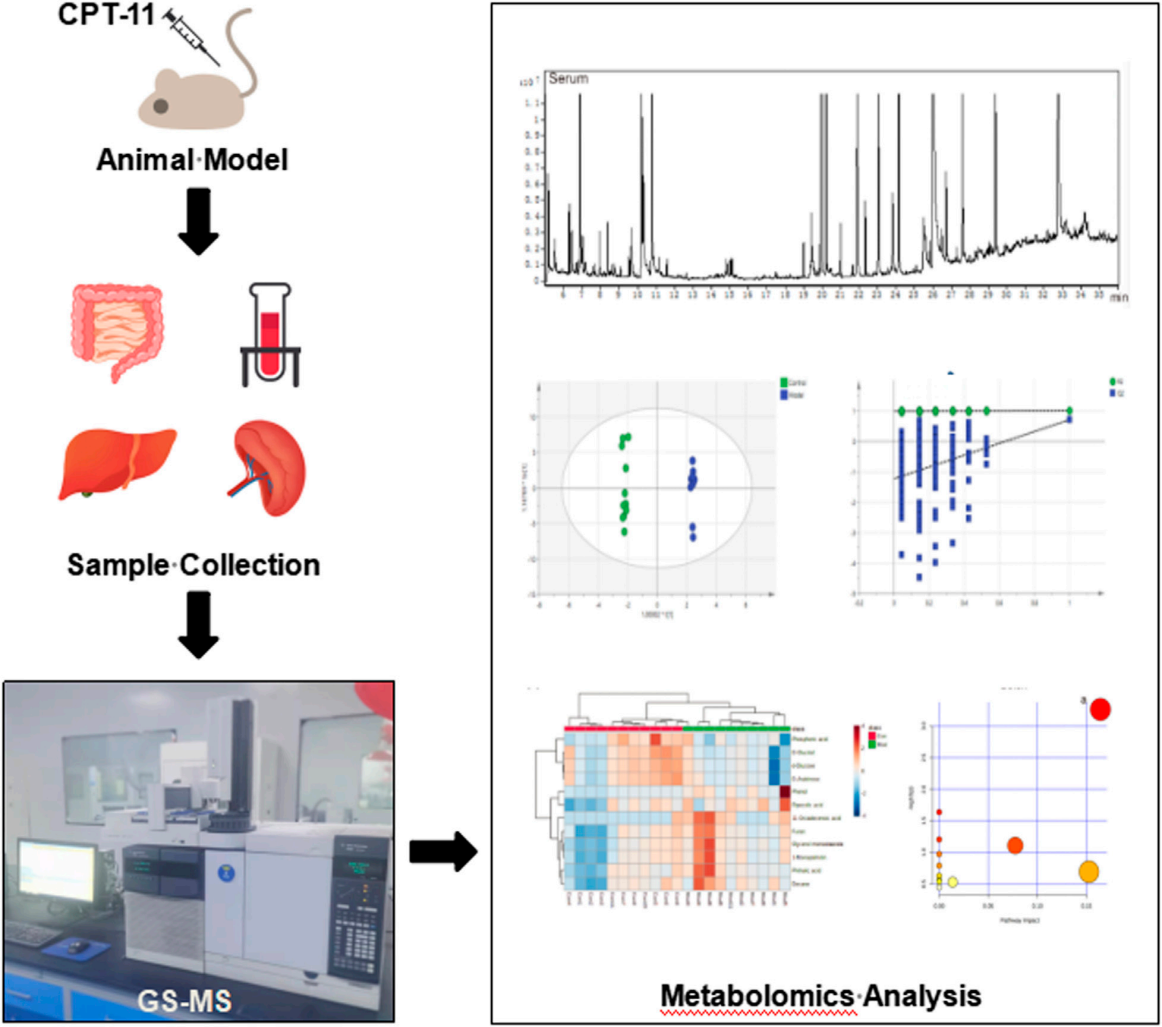
The workflow of the study. The male ICR mice were treated with CPT-11 (20 mg/kg daily; i.p.) to establish the IIM animal model. On the 9th day after administration, the serum, intestinal, colonic, hepatic, and splenic samples were collected. Gas chromatography–mass spectrometry (GC–MS) was used to investigate the metabolic alterations in the main tissues of the animal model. Multivariate statistical analyses, including GC–MS data processing, pattern recognition analysis, and pathway analysis, were performed.

Metabolomic analysis of inflammatory bowel disease (IBD) has been studied extensively (Kostic et al., 2014; Lloyd-Price et al., 2019; Baier et al., 2020); however, that of irinotecan-induced intestinal mucositis (IIM) has not been studied extensively. In this study, we investigated the metabolic alterations of serum and tissues from the intestine, colon, liver, and spleen of mouse models of IIM. Using GC-MS, metabolomic analyses revealed the metabolic pathogenesis of IIM to provide new options for diagnosis and treatment. The schematic illustration of the study design is shown in Figure 1.

## Materials and methods

### Ethical approval of the study protocol

The study protocol was approved by the Ethics Committee of Jining First People’s Hospital (No. JNMC-2021-DW-044, Jining, China). Efforts were made to minimize animal distress and the number of mice used. Animal care and husbandry were performed in strict accordance with the recommendations from the *Guide for the Care and Use of Laboratory Animals* (Ministry of Science and Technology of China, Beijing, China, 2006).

### Establishment of IIM mouse models

Male ICR mice (6–8 weeks old; Jinan Pengyue Laboratory Animal Breeding, Jinan, China) were used to investigate IIM based on our previous study (Yu et al., 2020). CPT-11 (Target Mol) was dissolved in sodium chloride containing 5‰ dimethyl sulfoxide (DMSO; Tianjin Yongda Chemical Reagents, Tianjin, China) and prepared to the required concentrations. Mice in the model group were administered CPT-11 (20 mg/kg daily; *i.p*.). Mice in the control group were injected with sodium chloride containing 5‰ DMSO and used as blank controls through daily intraperitoneal injection. The body weight of mice in each group was recorded daily. The mice were carefully monitored to identify symptoms, such as diarrhea and bloody stools. On the 9th day after unilateral eyeball removal, blood was collected from all mice. Thereafter, vertebrae were dislocated to collect intestinal, colonic, hepatic, and splenic tissues from all animals.

### DAI calculation and histology

DAI is the combined score of weight loss (compared to initial weight), stool consistency, and bleeding. Scores are defined as follows: weight loss: 0 (no loss), 1 (1–5%), 2 (5–10%), 3 (10–20%), and 4 (>20%); stool consistency: 0 (normal), 2 (loose stool), and 4 (diarrhea); and bleeding: 0 (no blood), 1 (hemoccult positive), 2 (hemoccult positive and visual pellet bleeding), and 4 (gross bleeding, blood around anus). Afterwards, formalin-fixed colonic tissues were subjected to hematoxylin and eosin (H&E) staining. The histological score was assessed, with 0–4 points attributed to each of the following parameters: 1) leukocyte infiltration; 2) vascular congestion and erosion; and 3) anabrosis of epidermal cells. The sum of each score was calculated as histological score.

### Sample preparation

The obtained intestinal, colonic, hepatic, and splenic samples were washed with phosphate-buffered saline (PBS, pH = 7.2) and rapidly frozen at −80°C until required for use. To obtain the serum, blood was collected and centrifuged (4,500 × g, 5 min). Thereafter, 100 μL serum and 350 μL methanol (containing IS100 μg/mL) or 50 mg tissue and 1 ml methanol (containing IS1 mg/mL) were mixed and centrifuged (14,000 rpm, 4°C, 10 min). The supernatants of the total mixture were transferred to new tubes (2.0 ml) and dried to completion under a gentle stream of nitrogen gas at 37°C in a shaking water bath. Additionally, 80 μL o-methyl hydroxylamine hydrochloride (15 mg/ml in pyridine) was added and mixed gently. The solution was incubated for 90 min at 70°C, and 100 μL of BSTFA with 1% TMCS was added to the solution, followed by 60 min of incubation at 70°C. The solution was then vortexed, centrifuged (14,000 rpm, 2 min, 4°C), and filtered through a 0.22-μm filter membrane before GC-MS analysis.

### GC–MS analysis

The quality control (QC) sample was prepared for each matrix by combining equal amounts of each control-model sample. The stability of retention time (RT) was evaluated using the RT of IS. Both the QC and experimental samples were analyzed by GC-MS using a 7000C-series mass spectrometer with a 7890B GC system (Agilent Technologies, United States). Samples were separated using an HP-5MS fused silica capillary column. Helium was used as carrier gas with a flow rate of 1 ml/min and a split ratio of 50:1. The temperature program for GC began at 60°C for 4 min, increased by 8°C/min until 300°C, and held for 5 min. The temperatures of the injection, transfer-line, and ion-source were 280, 250, and 230°C, respectively. Electron impact ionization (−70 EV) of 20 spectra/s was used in the MS setting. MS detection was performed by electrospray ionization (ESI) in full scan mode with 50–800 mass/charge (*m/z*) values.

### Multivariate statistical analysis

MassHunter Qualitative Analysis (Agilent Technologies) was used for GC-MS data processing. Peaks were identified by the chromatographic deconvolution tool in MassHunter. The mass and compound filters were adjusted to ensure that 1–300 components were identified by the software. Then, metabolites were identified by matching the secondary mass spectra of compounds with NIST14.0 mass spectrometry library. Pattern recognition analysis (principal component analysis, PCA; orthogonal partial least squares discriminant analysis, OPLS-DA) of normalized data was performed using SIMCA-P 14.0 (Umetrics, Umea, Sweden). Two-tailed Student’s *t-test* was used to analyze data. The differential variables were selected based on these conditions: 1) *p* < 0.05 and 2) VIP value obtained from OPLS-DA >1. Pathway analysis was performed using MetaboAnalyst 5.0 (http://www.metaboanalyst.ca) and the Kyoto Encyclopedia of Genes and Genomes database. The pathways for raw *p* < 0.05 and impact >0 were considered significant. These metabolomic analytical methods were used in our previous studies (Geng et al., 2021; Zhao et al., 2021).

## Results

### CPT-11 administration could cause IIM in ICR mice

**FIGURE 2:**
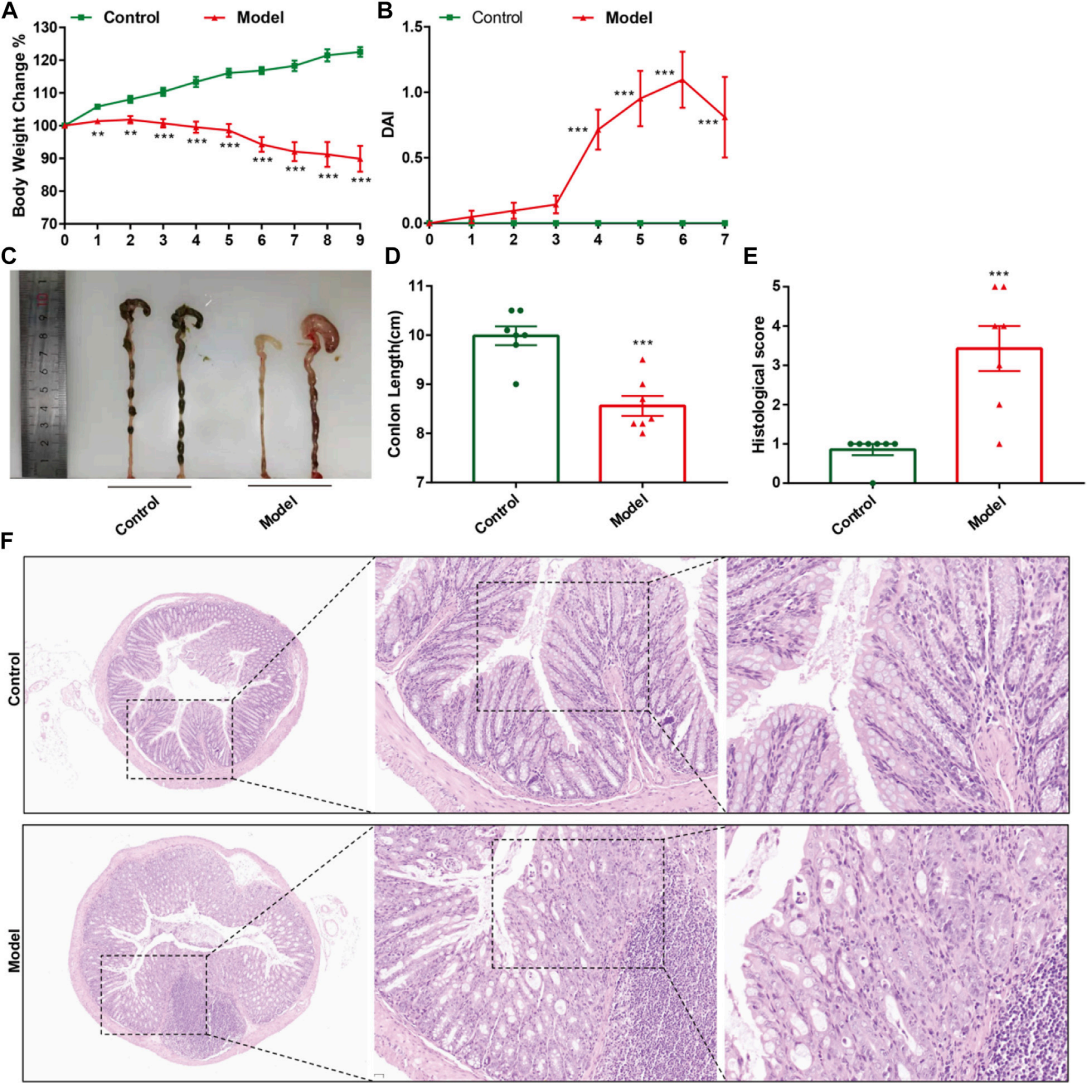
CPT-11 administration could lead to IIM in ICR mice. Mice were injected (i.p.) with CPT-11 (20 mg/kg, daily) for 9 days before being killed. **(A)** Body weight changes. **(B)** The DAI score. **(C, D)** Macroscopic images and length of the colon. **(E)** Histopathology score of colonic tissue. (*n* = 7) **p < 0.01, ***p < 0.001 vs. Control, *t*-test. **(F)**. Colonic tissues stained with H&E, and the photos were observed by confocal laser-scanning microscope, 50×, 200×, 400×.

CPT-11 was used to establish mouse models of IIM. Changes in body weight, DAI score, colorectal shortening, and H&E staining of the intestinal mucosa were used to evaluate IIM. CPT-11 administration reduced the body weight of mice (Figure 2A) and increased DAI score (Figure 2B). To observe intestinal lesions in mice with IIM, the colorectal length was measured 9 days after CPT-11 administration. CPT-11 shortened the colon length significantly (Figures 2C,D). Thereafter, intestinal samples were collected and stained by H&E staining. Colonic damage (necrosis of the intestinal mucosa, infiltration of inflammatory cells, submucosal edema, and ulcer formation) was detected in mice (Figures 2E,F).

### GC-MS chromatograms of QC samples in IIM

**FIGURE 3:**
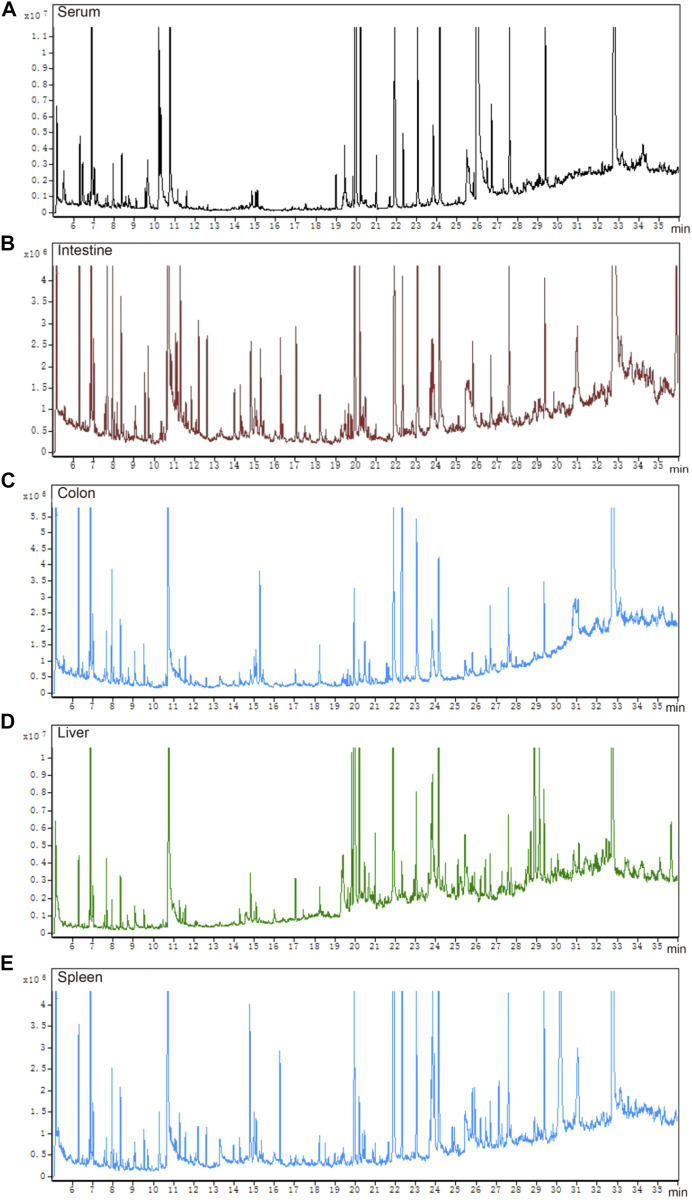
Total ion chromatograms of QC samples. **(A)** serum, **(B)** intestine, **(C)** colon, **(D)** liver, and **(E)** spleen.

The total ion chromatograms of QC samples, including serum (Figure 3A), intestine (Figure 3B), colon (Figure 3C), liver (Figure 3D), and spleen (Figure 3E), were obtained by optimizing the detection conditions of GC-MS. Most of the peaks in the total ion chromatograms were separated at baseline (Figure 3). This observation showed that the peak area of the internal standard was stable, and that the retention time was identical. All total ion chromatograms showed strong signals and good reproducibility.

### Multivariate statistics

**FIGURE 4:**
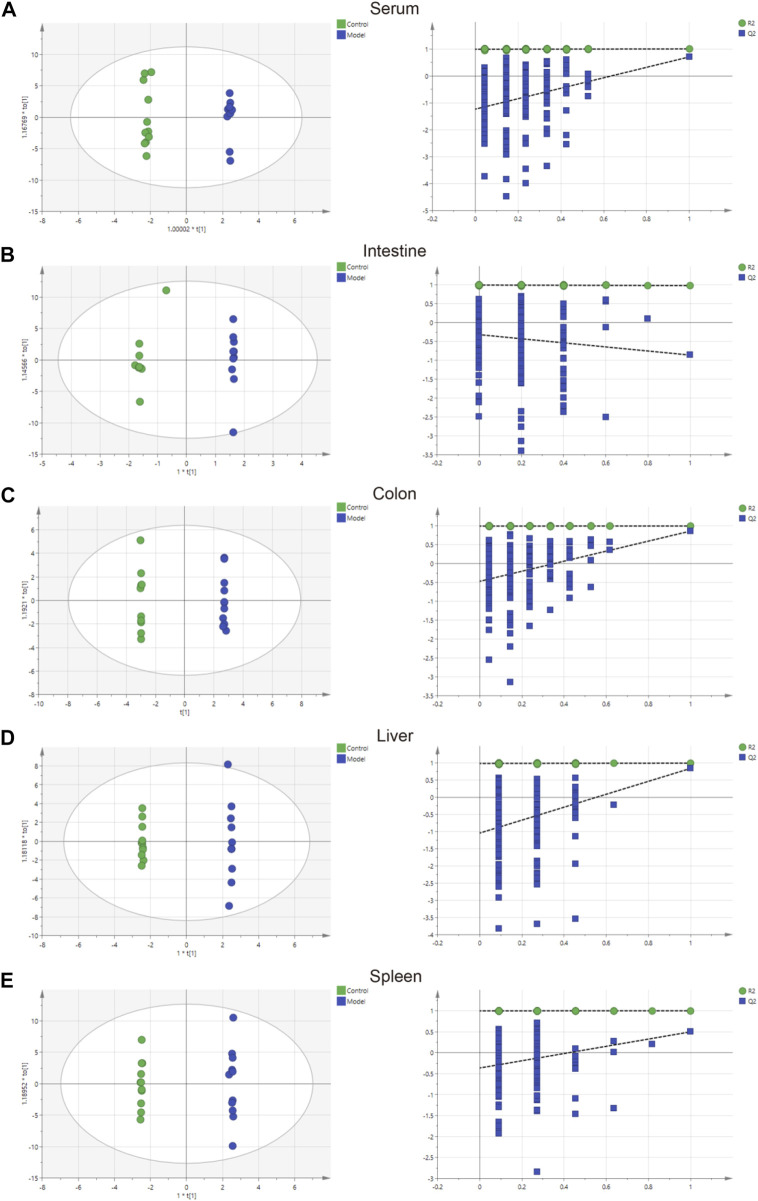
Charts showing the OPLS-DA score and 200 permutation tests. **(A)** serum, **(B)** intestine, **(C)** colon, **(D)** liver, and **(E)** spleen.

**FIGURE 5:**
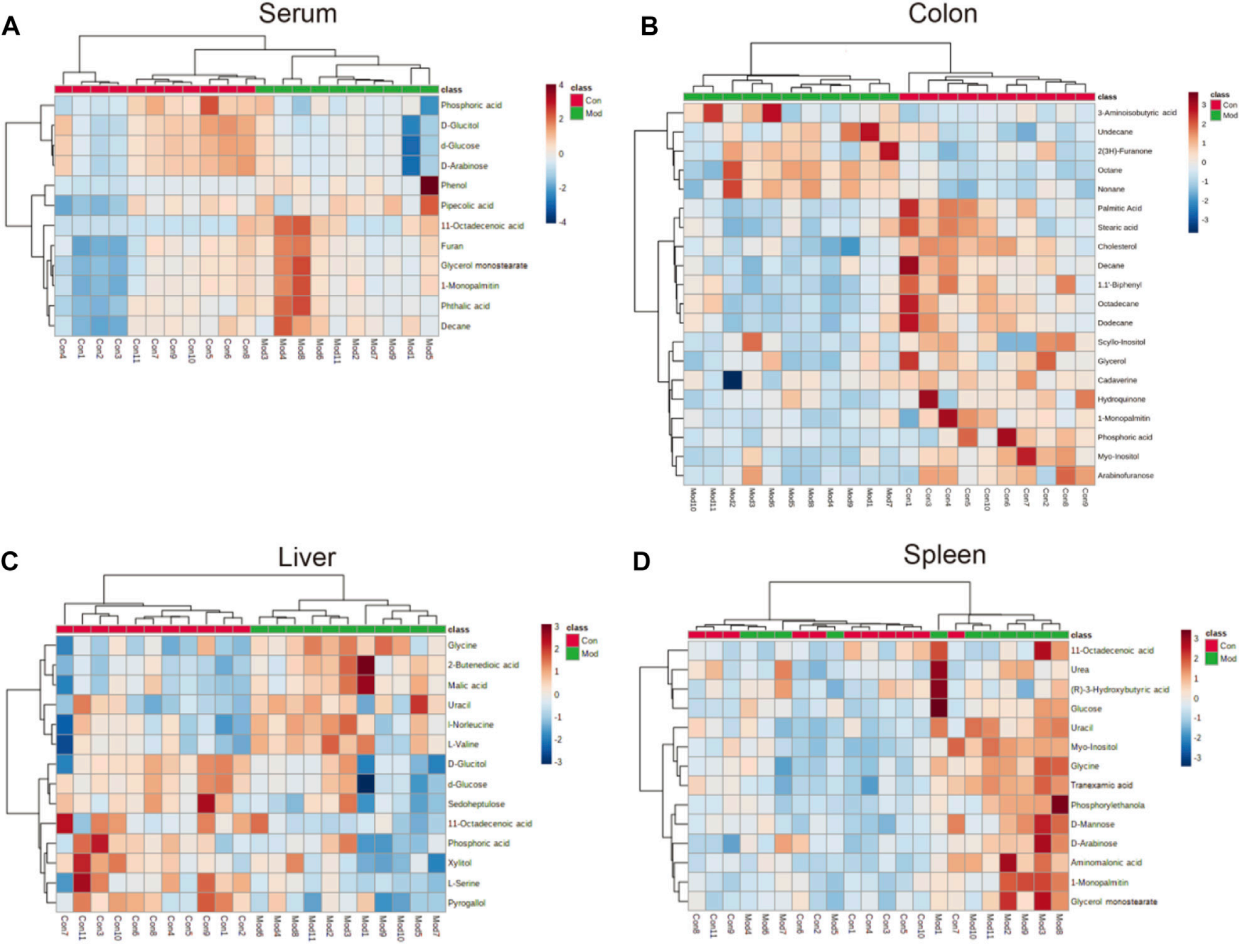
Heatmap of different metabolites in the **(A)** serum, **(B)** colon **(C)** liver, and **(D)** spleen in the model group compared with the control group. The color of each part represents the importance of metabolite changes (blue = downregulated; red **=** upregulated). Rows represent samples. Columns represent metabolites.

The parameters of PCA indicated efficient modeling of IIM that clearly separated the control and model groups (serum: R2X = 0.924, R2Y = 0.998, Q2 = 0.705; intestine: R2X = 0.845, R2Y = 0.938, Q2 = −0.895; colon: R2X = 0.773, R2Y = 1, Q2 = −0.865; liver: R2X = 0.867, R2Y = 0.999, Q2 = 0.847; spleen: R2X = 0.825, R2Y = 1, Q2 = 0.509). These parameters approaching 1.0 indicate that the IIM model was stable and predictably reliable. Statistical validation using OPLS-DA revealed no overfitting (the blue regression line of the Q2-points intersects the vertical axis on the left below zero; all Q2-values on the left were lower than the original points on the right) in serum and tissue samples, except intestine (Figure 4). Moreover, cluster analyses on metabolite expression in serum and tissue samples (colon, liver, and spleen) of each group are shown in Figure 5. Compared with metabolites of the control group, metabolites of the model group were significantly different in serum, colon, and liver. These results implied that the experiment was reproducible and the data were reliable.

### Identification of potential biomarkers

Forty-six metabolites with significant changes in level in tissues and serum were identified by Student’s *t*-test (*p* < 0.05) and VIP score (VIP >1) screening between the control and model groups. In the model group, 11 metabolites were identified in serum; most of which were hydrocarbons, lipids, benzenoids, and heteroaromatic compounds. Several metabolites were identified in the colon, with 20 metabolites, including hydrocarbons, amino acids, lipids, benzenoids, and amines, showing differences in level. Fourteen metabolites, including carbohydrates, amino acids, benzenoids, and hydroxy acids, had different levels in the liver. The spleen contained 12 metabolites, including carbohydrates, amino acids, lipids, and pyridines, at different levels (Table 1).

**TABLE 1:** Metabolites with changes in the serum, intestine, colon, liver, and spleen.

Metabolites	HMDB	Serum		Intestine		Colon		Liver		Spleen	
		VIP	Trend	VIP	Trend	VIP	Trend	VIP	Trend	VIP	Trend
(R)-3-Hydroxybutyric acid	HMDB0000011									1.46	↑
1,1'-Biphenyl	HMDB0034437					1.85	↓				
11-Octadecenoic acid	HMDB0003231	1.81	↑					1.33	↓		
1-Monopalmitin	HMDB0011564	1.36	↑			1.26	↓			1.61	↑
2(3H)-Furanone	HMDB0094707					1.33	↑				
2-Butenedioic acid	HMDB0000176							1.93	↑		
3-Aminoisobutyric acid	HMDB0003911					1.17	↑				
Aminomalonic acid	HMDB0001147									1.78	↑
Arabinofuranose	HMDB0012325					1.33	↓				
Cadaverine	HMDB0002322					1.35	↓				
Cholesterol	HMDB0000067					1.84	↓				
D-Arabinose	HMDB0029942	1.46	↓							1.94	↑
Decane	HMDB0031450	1.30	↑			1.40	↓				
D-GluCIMol	HMDB0000247	1.64	↓					1.97	↓		
D-Mannose	HMDB0000169									1.47	↑
Dodecane	HMDB0031444					1.52	↓				
Furan	HMDB0013785	1.31	↑								
Glucose	HMDB0000122	1.58	↓					1.79	↓	2.19	↑
Glycerol	HMDB0000131					1.24	↓				
Glycerol monostearate	HMDB0011535	1.46	↑							1.42	↑
Glycine	HMDB0000123							2.00	↑	1.86	↑
Hydroquinone	HMDB0002434					1.22	↓				
l-Norleucine	HMDB0001645							1.52	↑		
L-Serine	HMDB0000187							1.49	↓		
L-Valine	HMDB0000883							1.61	↑		
Malic acid	HMDB0000744							1.85	↑		
Myo-Inositol	HMDB0000211					1.77	↓			1.63	↑
Nonane	HMDB0029595					1.61	↑				
Octadecane	HMDB0033721					1.54	↓				
Octane	HMDB0001485					1.61	↑				
Palmitic Acid	HMDB0000220					1.78	↓				
Phenol	HMDB0000228	1.39	↑								
Phosphoric acid	HMDB0002142					1.34	↓	1.25	↓		
Phosphorylethanolamine	HMDB0000224									1.61	↑
Phthalic acid	HMDB0002107	1.34	↑								
Pipecolic acid	HMDB0000070	1.30	↑								
Pyrogallol	HMDB0013674							1.78	↓		
Scyllo-Inositol	HMDB0006088			2.21	↑	1.48	↓				
Sedoheptulose	HMDB0003219							1.44	↓		
Stearic acid	HMDB0000827					1.37	↓				
Tranexamic acid	HMDB0014447									1.46	↑
Undecane	HMDB0031445					1.24	↑				
Uracil	HMDB0000300							1.75	↑	1.80	↑
Urea	HMDB0000294									1.44	↑
Xylitol	HMDB0002917							1.50	↓		

### Analyses of metabolic pathways

**FIGURE 6:**
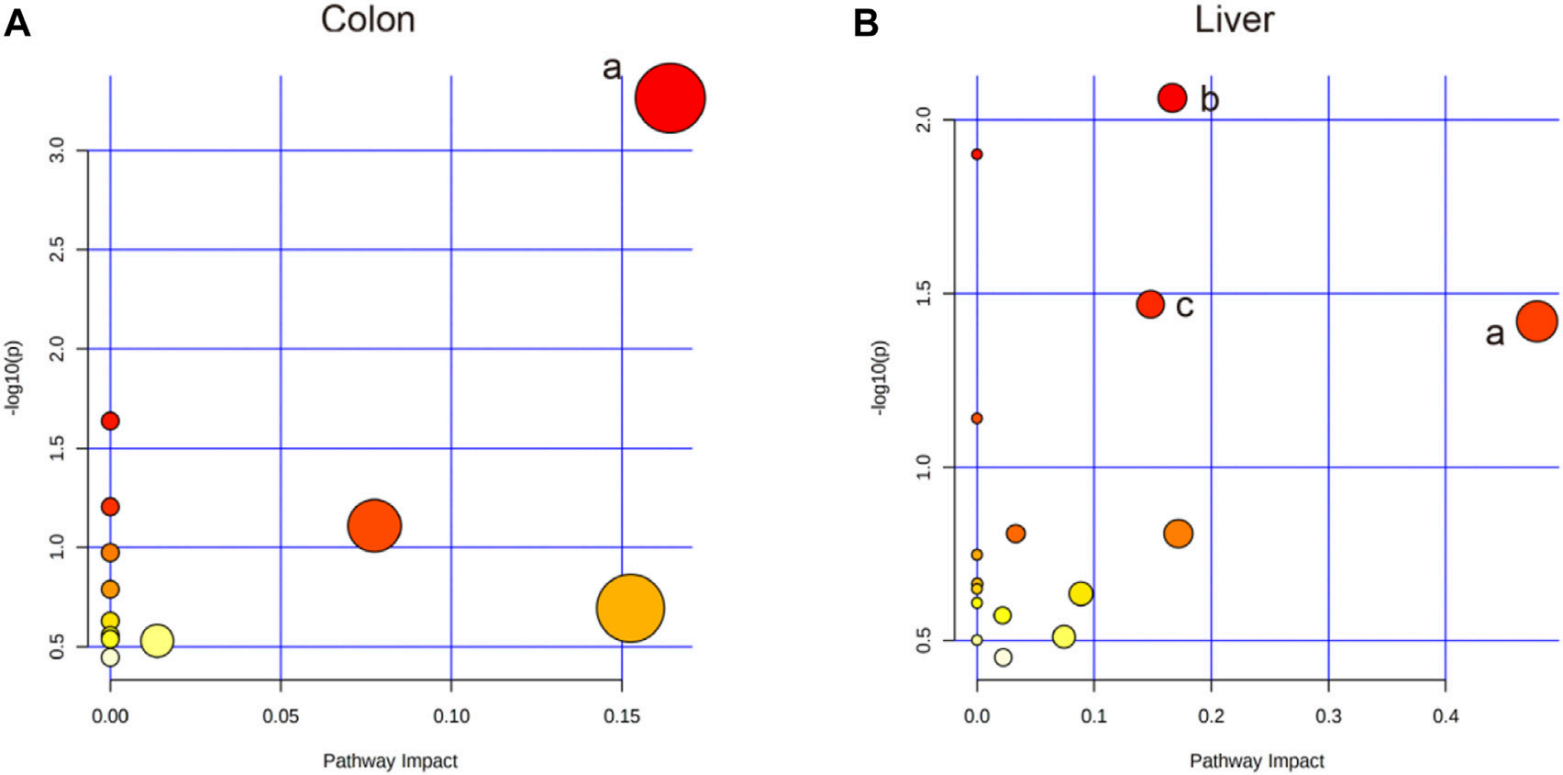
Pathway analysis using MetaboAnalyst™ 5.0. **(A)** The colon: a. Glycerolipid metabolism. **(B)** The liver: a. Glycine, serine, and threonine metabolism; b. Aminoacyl-tRNA biosynthesis; c. Glyoxylate and dicarboxylate metabolism.

Based on the profiles of annotated metabolites, metabolic pathway analyses were performed to reveal the most relevant pathways related to IIM. Four metabolic pathways, glycerolipid metabolism in the colon and aminoacyl-tRNA biosynthesis; glyoxylate and dicarboxylate metabolism; and glycine, serine, and threonine metabolism in the liver were significantly different (raw *p* < 0.05 and impact >0), as shown in Table 2 and Figure 6.

**TABLE 2:** Metabolic pathways related to IIM.

Pathway name	Tissue	Match status	Raw *p*	Impact
Glycerolipid Metabolism	Colon	3/23	1.07E-02	0.164
Aminoacyl-tRNA biosynthesis	Liver	3/48	8.643E-03	0.167
Glyoxylate and dicarboxylate metabolism	Liver	2/19	3.395E-02	0.148
Glycine, serine and threonine metabolism	Liver	2/34	3.800E-02	0.478

## Discussion

This study is the first to use metabolomics analysis for systematic evaluation of IIM. Metabolomic alterations in the main tissues (serum, intestine, colon, liver, and spleen) of mice were systematically profiled, revealing 11, 20, 14, and 12 metabolites in the serum, colon, liver, and spleen of mice, respectively, between the model and control groups (Table 1). The disturbances in the identified metabolites were mainly involved in the following pathways: glycerolipid metabolism, aminoacyl-tRNA biosynthesis, glyoxylate and dicarboxylate metabolism, and glycine, serine, and threonine metabolism (Table 2 and Figure 6).

CIM has three main pathologic mechanisms: inflammatory reaction (Davis 2000; Angel, Szabowski, and Schorpp-Kistner 2001; Bamba et al., 2003), gut-flora imbalance (Alimonti et al., 2004; Alexander et al., 2017) and ischemia (Yu et al., 2020). Drug metabolism is a special pathologic mechanism of IIM. Previous studies focused on the liver-intestinal circulation pathway of CPT-11 but neglected the effect of CPT-11 on metabolism in major organs leading to IIM (Mathijssen et al., 2001; de Man et al., 2018; Zhu et al., 2020). Our study provided the first comprehensive and systematic view of metabolic alterations of IIM. Many of the metabolites and their related pathways identified could be candidate factors for IIM development, and they may be potential biomarkers for diagnosis and treatment.

### Inflammatory reaction-related metabolic changes

11-Octadecenoic acid (vaccenic acid [VA]) is an anti-inflammatory factor. VA is involved in the pathophysiologic mechanism of several diseases (Wang et al., 2012; Jacome-Sosa et al., 2016; Sulijaya et al., 2018). VA activates peroxisome proliferator-activated receptors (PPARs) in the intestines and mediates anti-inflammatory effects by antagonizing the actions of a pro-inflammatory transcription factor: nuclear factor-kappa B (NF-κB). This action leads to downregulation of expression of pro-inflammatory markers, such as interleukin-1β (IL-1β), tumor necrosis factor-α (TNF-α), and IL-12 (Daynes and Jones 2002; Loscher et al., 2005). Moreover, expression of IL-1β and TNF-α is upregulated *via* NF-κB signaling (Logan et al., 2008; Logan et al., 2008), which occurs during the early stage of CIM and induces mucosal injury not only by direct damage to tissue but also by providing a positive-feedback loop to amplify the primary damage (Davis 2000; Angel et al., 2001; Bamba et al., 2003). The VA level was reduced in the livers of mice in the model group (Figure 5C), which could aggravate the inflammation caused by IIM. However, the VA level was increased in serum (Figure 5A). Additionally, L-Serine reduces the secretion of pro-inflammatory cytokines, including IL-1, IL-17, interferon-γ, and TNF-α, in serum to inhibit macrophage- and neutrophil-mediated inflammatory responses (He et al., 2019). L-Serine level was reduced in the livers of mice in the model group (Figure 5C), similar to the observation on the VA level.

Although some studies have indicated that pyrogallol has anti-inflammatory actions (Nicolis et al., 2008; Geißler et al., 2019), other studies have refuted this hypothesis (Sharma et al., 2000; Upadhyay et al., 2010). The pyrogallol level in the liver was reduced (Figure 5C), indicating that pyrogallol may be protective against IIM. Stearic acid can function as a natural ligand of PPARs and protect cells from oxidative damage (Wang et al., 2007). Stearic acid can attenuate bile duct ligation (BDL)-induced inflammation by suppressing recruitment or accumulation of inflammatory cells and NF-κB activation (Pan et al., 2010). The level of stearic acid in the colon was reduced, indicating that stearic acid may be a protective metabolite against IIM (Figure 5B).

### Imbalance in gut flora-related metabolic changes

Furan can trigger an imbalance in intestinal flora (e.g., bacteria of the class Clostridia and *Lactobacillus* species) (Yuan et al., 2019). A similar gut-flora imbalance has been noted in CIM (Alimonti et al., 2004; Alexander et al., 2017). The furan level was increased in the serum of IIM mice (Figure 5A). L-Serine is another metabolite that increases bacterial fitness and provides *Enterobacteriaceae* with a growth advantage against their competitors in the inflamed gut (Kitamoto et al., 2020). This is a contradiction of its anti-inflammation of CIM, as already mentioned in.

### Ischemia-related metabolic changes

Hydroquinone is an inhibitor of lipid peroxidation. Due to its reactivity with lipoperoxy radicals and its ability to donate intramolecular hydrogen bonds, it has protective activity that transforms toxic reactive oxygen species (ROS) into nontoxic species (Roginsky et al., 2003; Rodríguez et al., 2007). Studies have shown that platelet activation is related to a significant increase in ROS number due to mitochondrial dysfunction (Geisler et al., 2007; Becatti et al., 2013; Caruso et al., 2015). In a previous study, we revealed the formation of intestinal microthrombi and different types of thrombi in CIM and found that thrombotic intestinal ischemic injury was a core trigger in the pathologic mechanism of IIM (Yu et al., 2020). In this study, the hydroquinone level was reduced in the colon, which could promote thrombotic intestinal ischemia in IIM. A recent study (Gu et al., 2019) conducted in patients with colorectal polyps revealed increased levels of lipids and reduced levels of glycerol, suggesting that glycerolipid metabolism was abnormal, which might participate in generation of adenosine triphosphate. Based on those data, glycerolipid metabolism with reduced levels of hydroquinone, glycerol, and palmitic acid was identified in the colons of IIM mouse models (Table 2).

### Drug metabolism-related metabolic changes

Arabinofuranose has been reported to be a component of polysaccharides that undergo activation during internal secretion (Zhou et al., 2007), osteogenesis (Zhao et al., 2014), and digestion (Golovchenko et al., 2012). Additionally, arabinofuranose may be a suitable carrier for drug delivery to hepatocytes (Groman et al., 1994). The elimination pathways of CPT-11 were mainly the hepatic metabolism and biliary secretion with major contributions from various enzymes, especially β-glucuronidases (Haaz et al., 1998; Mathijssen et al., 2001). Arabinofuranose level was reduced in the colons of mice in the model group (Figure 5B). This phenomenon probably affects absorption during digestion or metabolism by the liver because CPT-11 increases drug toxicity to induce IIM. Furan is another metabolite affecting hepatic metabolism by dysregulating the biosynthesis of primary bile acids (Yuan et al., 2020) and injury due to free radical-mediated lipid peroxidation (Yuan et al., 2013), which was increased to promote IIM (Figure 5A).

### Changes in other metabolites

Tranexamic acid has been shown to ameliorate ulcerative colitis in clinical studies (Hollanders et al., 1982; Almer et al., 1992). The underlying mechanism and its influence on CIM are controversial. Moreover, our findings on metabolite levels in the spleens of IIM mouse models (Figure 5D) were contradictory. Glycine has been shown to be a protective factor against gastrointestinal diseases, such as chemical-induced colitis (Tsune et al., 2003) and IBD (Chou et al., 2014; Turer et al., 2017). However, glycine level was increased (Figure 5C), indicating that glycine may have a protective role in IIM.

## Conclusion

Metabolomic changes in the serum and tissues (colon, intestine, liver, and spleen) of mice after IIM induction were analyzed using GC-MS. The results provided a holistic view of metabolic alterations of hydrocarbons, amino acids, lipids, benzenoids, hydroxy acids, and amines involved in carbohydrate, amino acid, and lipid metabolism in IIM-associated tissues. The characterization of metabolomic patterns (especially in colonic and hepatic samples) and relationship between the metabolites and metabolic pathways could provide potential diagnostic clues for further research and profound theoretical understanding of IIM pathogenesis.

## Data Availability

The original contributions presented in the study are included in the article/supplementary material, further inquiries can be directed to the corresponding author.
